# Validation of Blood Pressure Measurement Using a Smartwatch in Patients With Parkinson's Disease

**DOI:** 10.3389/fneur.2021.650929

**Published:** 2021-06-29

**Authors:** Jong Hyeon Ahn, Joomee Song, Inyoung Choi, Jinyoung Youn, Jin Whan Cho

**Affiliations:** ^1^Department of Neurology, Samsung Medical Center, Sungkyunkwan University School of Medicine, Seoul, South Korea; ^2^Neuroscience Center, Samsung Medical Center, Seoul, South Korea

**Keywords:** wearable device, blood pressure, Parkinson's disease, photoplethysmography, validation, sphygmomanometer

## Abstract

**Objectives:** We aimed to validate the accuracy of blood pressure (BP) measurement using a smartwatch in patients with Parkinson's disease (PD).

**Materials and Methods:** We compared 168 pairs of BP (*n* = 56) measurements acquired by a smartwatch (SM-R850) with those measured by a sphygmomanometer (reference device).

**Results:** Differences between the smartwatch BP and reference BP measurements were compared. The mean and standard deviation of the differences systolic BP (SBP) and diastolic BP (DBP), measured by smartwatch and reference device, fulfilled both criterion 1 (0.4 ± 4.6 and 1.1 ± 4.5 mm Hg for DBP and SBP, respectively) and criterion 2 (0.2 ± 2.5 and 0.9 ± 2.4 mm Hg for DBP and SBP, respectively) of the BP validation criterion of the International Organization for Standardization.

**Conclusion:** BP measurement using a smartwatch with a photoplethysmography sensor is an accurate and reliable method in patients with PD.

## Introduction

Autonomic dysfunction is highly prevalent in patients with Parkinson's disease (PD) (1). A study reported that 32% of patients with PD had orthostatic hypotension (OH), which is associated with a poorer prognosis, cognitive decline, and a higher risk of falls, and it is an essential non-motor symptom to distinguish PD from multiple system atrophy (2, 3). Supine hypertension (SH) is also common in PD and induces end-target organ damage, such as cerebrovascular damage, heart disease, and nephropathy (4). Early detection and management of these blood pressure (BP) fluctuations may help to reduce cardiovascular risk and the risk of falls, and improve the quality of life of patients with PD (5).

Regular and repetitive measurement of the BP to detect fluctuations in BP is crucial for the management and diagnosis of PD (3, 6). Recently, cuff-less wristwatch-type devices that can measure the BP using photoplethysmography (PPG) sensors have been introduced (7). The measurement of the BP with a smartwatch facilitates BP monitoring in patients anytime and anywhere; however, patients with PD have been excluded from validation studies because they were believed to be subjects not suitable for the validation of BP measurement using a PPG sensor because of involuntary movement (8, 9), despite that the validation of the measurement of the BP using a smartwatch should be preceded for future studies, and clinical practice of PD. Therefore, in the present study, we validated the accuracy and reliability of BP measurement using a smartwatch in patients with PD.

## Methods

### Participants

Patients with PD were recruited from the Department of Neurology, Samsung Medical Center, from October 2020 to November 2020. The diagnosis of PD was made using the United Kingdom Parkinson's Disease Brain Bank criteria (10). The inclusion criteria were as follows: age between 40 and 80 years, with normal cognition [Mini-Mental State Examination (MMSE) ≥ 26] who can understand informed consent. The exclusion criteria were as follows: atrial or ventricular arrhythmias, pacemaker, heart disease, or vascular disease on the arms, musculoskeletal disease that may prevent BP measurement, and a difference in systolic BP (SBP) or diastolic BP (DBP) of >10 mm Hg between the two arms, as measured by a sphygmomanometer. We also excluded patients who had severe involuntary movement including those with dyskinesia and/or resting tremor on the less affected side [resting tremor score ≥2 by the United Parkinson's Disease Rating Scale (UPDRS)] (11). In terms of mild dyskinesia, if the BP was correctly measured without any alarm or warning sign on the test device, the patients were included despite that the patients had mild dyskinesia. We collected demographic and medical information of the participants, including age, sex, body mass index (BMI), disease duration, UPDRS III (evaluated in medication “on” state), Hoehn and Yahr (H & Y) stage, MMSE, levodopa equivalent dose (LED), and comorbidities (12, 13). This study was approved by the institutional review board of Samsung Medical Center, and all subjects provided written informed consent.

### Test Device

An SM-R850, 41 mm (Samsung Electronics, Suwon, Gyeonggi, South Korea), a smartwatch released on August 6, 2020, in South Korea, was used for BP measurement. The device uses a light-emitting diode PPG sensor for the measurement of the BP and was approved as a medical device by the Korean Ministry of Food and Drug Safety on April 20, 2020. The SM-R850 was calibrated with an aneroid sphygmomanometer (reference device, ri-san® Rudolf Riester, Jungingen, Germany) prior to BP measurement. Three pairs of smartwatch and reference BP measurements were obtained for each participant.

### Calibration of the SM-R850 and BP Measurements Using the SM-R850 and the Sphygmomanometer

Calibration of the tested device was conducted using the following steps, as recommended by the manufacturer: (1) the observer ensured that the smartwatch was securely fastened 2 cm proximal from the participant's less affected side wrist; (2) the observer fit the BP cuff on the opposite-side arm of the participant and started to measure the BP using a sphygmomanometer; (3) the watch captured the BP of the participant, and the measured BP was entered using a sphygmomanometer on the smartphone application (Samsung Health Monitor; Samsung Electronics, Suwon, Gyeonggi, South Korea). If the watch was unable to correctly capture the participant's BP, the same protocol was attempted once more. (4) The participants were asked to remain still and not talk while the BP was being measured by the watch. None of these measurements were used to calculate the accuracy of the smartwatch.

A trained observer measured the reference BP using aneroid sphygmomanometers and a “Y” connected teaching stethoscope (3M Littmann Classic III; 3M Health Care, St. Paul, MN, USA). Korotkoff K1 was used for the reference SBP and K5 for the DBP in all subjects. Three cuff sizes were used according to the mid-arm circumference: a small cuff for arm circumferences of 18–23 cm; a standard cuff (medium), 24–32 cm; and a large cuff, 32–42 cm.

The test BP using a smartwatch was measured on the less affected side wrist in sitting position, between 9 a.m. and 2 p.m., after at least 5 min of rest. The BP measurement was performed in the medication “on” state. The reference measurement of the BP was also measured simultaneously at the level of the heart on the opposite-side arm of the participants according to the guideline of the manufacturer, and the same protocol was repeated three times with at least 1 min of interval between each measurement. Each smartwatch measurement was compared with the reference measurement, which was taken simultaneously with the smartwatch.

### Statistical Analyses

Data were analyzed according to two criteria required by the International Organization for Standardization (ISO), which is designed to provide 98% confidence interval and a statistical power of 95% (14). For criterion 1, we compared each SBP and DBP measured by the smartwatch and the reference device, and the mean error of all valid paired BP should be ≤ 5 mm Hg with a standard deviation (SD) <8 mm Hg. The maximum permissible SD is calculated based on the mean difference of criterion 1. Bland–Altman plots were used to show deviations in the data. Pearson correlation analysis was performed to investigate the correlation between the BP measured by each device. The association between the BP and clinical features of the participants was compared using Spearman rank order correlation analysis. Statistical analyses were performed using IBM for Windows (version 25.0; IBM Inc., Armonk, NY, USA).

## Results

**Figure 1:**
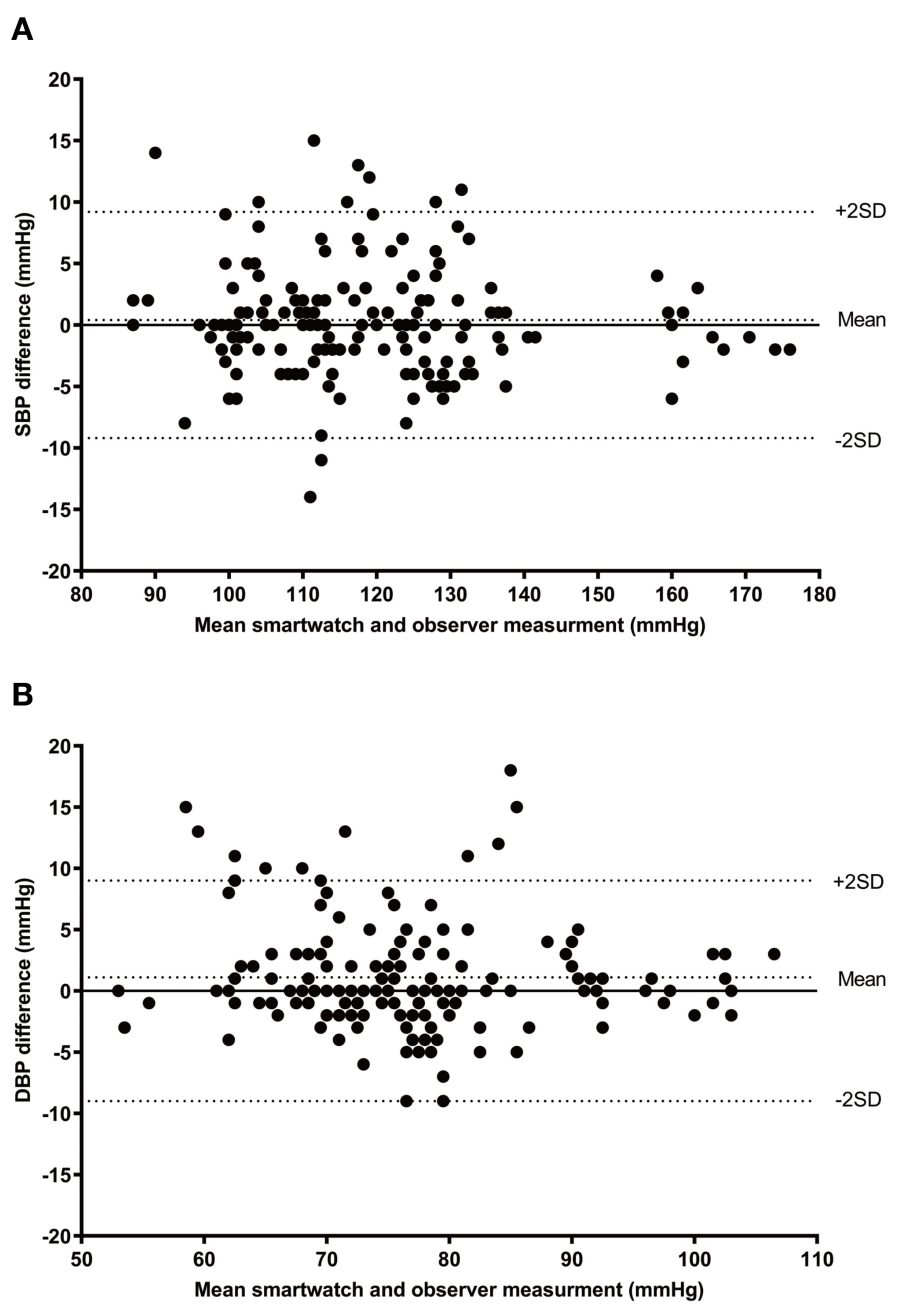
Bland–Altman plots for the differences between the SM-R850 readings and the reference measurements for SBP **(A)**, and DBP **(B)** (*n* = 168). SBP, systolic blood pressure; DBP, diastolic blood pressure.

Sixty-two participants were screened, and six of them were excluded for the following reasons: two because of arrhythmia, one with a difference in BP > 10 mm Hg between the two arms, and three were not included in the final analysis because they failed to obtain three pairs of BP measurements using the test device because of resting tremor. Therefore, 56 participants were enrolled and included for the final analysis in the study. The characteristics and demographics of the participants are described in Table 1. We obtained a total of 168 pairs of measurements of the smartwatch and reference SBP and DBP. The mean SBP and DBP measured by smartwatch were 118.9 mm Hg (range = 87–175 mm Hg) and 77.5 mm Hg (range = 52–108 mm Hg), respectively. The mean SBP and DBP of the reference device were 118.6 mm Hg (range = 83–177 mm Hg) and 76.3 (range = 51–105 mm Hg), respectively. Bland–Altman plots of SBP and DBP differences between the smartwatch and reference measurements with 168 pairs are given in Figure 1. The mean and SD of the differences between the smartwatch and reference SBP and DBP measurements fulfilled both criterion 1 (0.4 ± 4.6 mm Hg for SBP and 1.1 ± 4.5 mm Hg for DBP), and criterion 2 (0.2 ± 2.8 mm Hg for SBP and 0.9 ± 2.4 mm Hg for DBP).

**Table 1:** Clinical characteristics and demographics of the study population.

**Variables**	**Values**
Age (years)	66.9 ± 9.1
Sex (male)	33 (58.9%)
Disease duration (month)	61.8 ± 51.6
BMI (kg/m^2^)	23.6 ± 3.3
No. of qualified participants	56
No. of BP pairs	168
UPDRS III (on)	17.8 ± 10.7
H & Y stage (on)	2.1 ± 0.9
MMSE	28.3 ± 1.2
LED (mg)	496.7 ± 317.3
Circumstance of arm (cm)	26.5 ± 2.8
SBP (proportion of measurements), mm Hg	
≥160	10 (6.0%)
≥140	15 (8.9%)
≤ 100	16 (9.5%)
DBP (proportion of measurements), mm Hg	
≥100	9 (5.3%)
≥85	32 (19.0 %)
≤ 60	10 (3.0%)
Heart rate (pulse/min)	76.4 ± 11.7

*Data are expressed as the number (%) or mean ± standard deviation. Fifty-six participants, with a total of 168 blood pressure measurements. BMI, body mass index; BP, blood pressure; UPDRS, United Parkinson's disease rating scale; H & Y, Hoehn and Yahr; MMSE, mini-mental state examination; LED, Levodopa equivalent dose; SBP, systolic blood pressure; DBP, diastolic blood pressure*.

The SBP and DBP measured by each device were highly correlated (SBP, *r* = 0.967, *p* < 0.001; DBP, *r* = 0.916, *p* < 0.001). The SBP measured by the test device was positively correlated with BMI (*r* = 0.432, *p* = 0.005), but not with age (*r* = −0.071, *p* = 0.658), disease duration (*r* = 0.028, *p* = 0.861), UPDRS III (*r* = 0.106, *p* = 0.511), H & Y stage (*r* = 0.114, *p* = 0.478), and LED (*r* = −0.159, *p* = 0.320). No correlation was found between DBP and clinical features, including age (*r* = 0.104, *p* = 0.517), disease duration (*r* = −0.061, *p* = 0.704), BMI (*r* = 0.170, *p* = 0.288), UPDRS III (*r* = 0.096, *p* = 0.552), H & Y stage (*r* = −0.090, *p* = 0.578), and LED (*r* = −0.194, *p* = 0.225).

## Discussion

This is the first study to validate BP measurement using a smartwatch (SM-R850) in patients with PD. Our study showed that using a smartwatch with a PPG sensor is an accurate and reliable method for measuring BP in patients with PD. Using the presented device, patients can measure their BP easily, regardless of time and place.

PPG sensors for assessing BP have been introduced as reliable and accurate tools. Recently, several cuff-less wristwatch-type BP-monitoring devices with PPG sensors have been introduced and have shown good accuracy (8, 15). However, patients with PD were excluded in a recent validation study (8) because they were considered as an inappropriate participant for the validation study due to involuntary movement such as resting tremor. The resting tremor is one of the cardinal symptoms of PD, and ~68% of PD patients had resting tremor, but it is much minor on the less affected side (16). In the present study, only fewer than 5% of the participants failed to measure the BP because of the resting tremor despite that the initial calibration was successfully done. The results of the study suggest that BP measurement using a smartwatch can be performed for PD patients when taking measurements on the less affected side and is reliable and accurate in PD patients as well.

The results of the study suggest that BP measurement using a smartwatch will become an important tool in monitoring of PD. In PD, 32% of patients had OH, but only one-third of them had symptoms associated with OH (2). This means that a substantial number of patients with PD and OH have not been realized or are not diagnosed in time (2). Furthermore, SH, which increases the risk of end-target organ damage, is also common in patients with PD (34%) (4, 17). The management of the BP in patients with PD is demanding because many patients have OH and SH simultaneously, and treatment of OH can induce SH and *vice versa*. Therefore, timely diagnosis, proper management, and monitoring of treatment-associated side effects of OH and SH are critical. A smartwatch is reproducible and able to monitor BP for a longer period than conventional ambulatory BP measurement devices, and it allows measurement of the BP whenever patients feel symptoms associated with OH, such as dizziness, faintness, and headache (6). In the current study, the measured SBP was positively correlated with BMI, which is consistent with the findings of a previous study reporting that lower BMI was associated with OH in PD (18). Maintaining a normal range of BMI may help to prevent the lowering of the BP or incidence of OH, and further research is warranted to confirm this finding.

This study has several limitations. First, the sample size was small, but it is acceptable, considering that the validation protocol recommended at least 35 participants for a special population (14). Second, ISO recommended the distribution of SBP and DBP, but the BP distribution of the enrolled participants did not fulfill the recommended range (14). Therefore, it should be carefully applied if the patients have high BP. Third, we excluded patients who had severe resting tremor at the very first step of the study, and it is still challenging to find an accurate and easier method to measure the BP in the PD population. Last, we calibrated the test device in the opposite arm according to the guideline of the manufacturer, and it can minimize the temporal variation of the BP. However, the experts recommend calibrating the smartwatch device using the same-arm measurement of the BP after acquiring PPG-based pulse signals in the smartwatch sequentially (19). The calibration in the same arm can be useful, especially for patients with PD because we can use the less affected arm for both the smartwatch and the reference device.

In conclusion, BP measurement using a smartwatch with a PPG sensor is accurate and reliable for patients with PD. The use of the device can be expanded to include detection and monitoring of the treatment response and side effects of OH and/or SH. Thus, we consider that smartwatches with PPG sensors will be highly valuable for management and future studies of PD.

## Data Availability Statement

The raw data supporting the conclusions of this article will be made available by the authors, without undue reservation.

## Ethics Statement

The studies involving human participants were reviewed and approved by Samsung Medical Center Instituitional Review Board. The patients/participants provided their written informed consent to participate in this study.

## Author Contributions

JHA and JWC: conceptualization, methodology, and writing—review and editing. JS, IC, and JHA: data curation. JHA: formal analysis and writing—original draft. JHA, JY, and JWC: investigation and resources. JWC: project administration and supervision. JS, IC, JWC, and JY: validation. All authors contributed to the article and approved the submitted version.

## Conflict of Interest

The authors declare that the research was conducted in the absence of any commercial or financial relationships that could be construed as a potential conflict of interest.
